# Protein structure shapes immunodominance in the CD4 T cell response to yellow fever vaccination

**DOI:** 10.1038/s41598-017-09331-w

**Published:** 2017-08-21

**Authors:** Maximilian Koblischke, Maria S. Mackroth, Julia Schwaiger, Ingrid Fae, Gottfried Fischer, Karin Stiasny, Franz X. Heinz, Judith H. Aberle

**Affiliations:** 1 Center for Virology, Medical University of Vienna, Vienna, Austria; 2 Department of Medicine I, Division of Infectious Diseases and Tropical Medicine, University Medical Center Hamburg-Eppendorf, Hamburg, Germany; 3 Department of Blood Group Serology and Transfusion Medicine, Medical University of Vienna, Vienna, Austria

## Abstract

The live attenuated yellow fever (YF) vaccine is a highly effective human vaccine and induces long-term protective neutralizing antibodies directed against the viral envelope protein E. The generation of such antibodies requires the help of CD4 T cells which recognize peptides derived from proteins in virus particles internalized and processed by E-specific B cells. The CD4 T helper cell response is restricted to few immunodominant epitopes, but the mechanisms of their selection are largely unknown. Here, we report that CD4 T cell responses elicited by the YF-17D vaccine are focused to hotspots of two helices of the viral capsid protein and to exposed strands and loops of E. We found that the locations of immunodominant epitopes within three-dimensional protein structures exhibit a high degree of overlap between YF virus and the structurally homologous flavivirus tick-borne encephalitis virus, although amino acid sequence identity of the epitope regions is only 15–45%. The restriction of epitopes to exposed E protein surfaces and their strikingly similar positioning within proteins of distantly related flaviviruses are consistent with a strong influence of protein structure that shapes CD4 T cell responses and provide leads for a rational design of immunogens for vaccination.

## Introduction

Yellow fever (YF) virus is a mosquito-borne member of the genus *Flavivirus*, family *Flaviviridae*, which includes other important human-pathogenic viruses, such as dengue (DEN), Zika (ZIK), West Nile (WN), Japanese encephalitis (JE) and tick-borne encephalitis (TBE) viruses^1^. A highly effective live attenuated vaccine (YF-17D) that confers protective immunity in almost all vaccinated individuals has been in use for many decades^2, 3^. The production of neutralizing antibodies (Abs) combined with CD4 T cell responses are critical for mediating such effective immunity^4, 5^. CD4 T cells provide help for Ab production through direct B-T cell interactions. These are mediated by the recognition of the same MHC II-bound peptides at the surface of B cells for which the T cells have been primed by dendritic cells (DCs). These B cell-presented peptides are derived from protein antigens that were internalized after specific recognition by the B cell receptor (BCR) and proteolytically processed by the B cells as antigen-presenting cells (APCs). In general, the CD4 T helper cell response is restricted to a few selected epitopes that dominate the response^6^. Mechanisms that control immunodominance and the selection of certain peptides are not entirely clear. Existing evidence suggests contributions of different factors, including affinity of peptide (p)MHC II complexes, the recognition of such complexes by the T cell receptor repertoire and structural features of the protein antigen that determine epitope selection during antigen processing in DCs^6–11^.

In the present study, we determined the specificities of the CD4 T cell responses in a cohort of 76 YF vaccinated persons and investigated to which extent immunodominance patterns correlated with structural features of virus proteins as well as *in silico* predicted peptide-MHC II affinities. A similar approach has recently been introduced for the distantly related flavivirus TBE virus^12^. The proteins of flaviviruses are structurally homologous but differ by up to 60% at the amino acid level. A comparison of the specificities in response to distantly related flaviviruses, such as TBE and YF viruses can thus exploit structural conservation and sequence divergence for studying the contribution of structural factors to immunodominance. High resolution structures have been obtained by X-ray crystallography and cryo-electron microscopy for structural proteins of several flaviviruses^13–22^, but not yet for YF virus. However, the available data indicate a high degree of structural conservation among all flaviviruses and it is therefore justified to assume that YF virus will have a similar structural organization.

**Figure 1:**
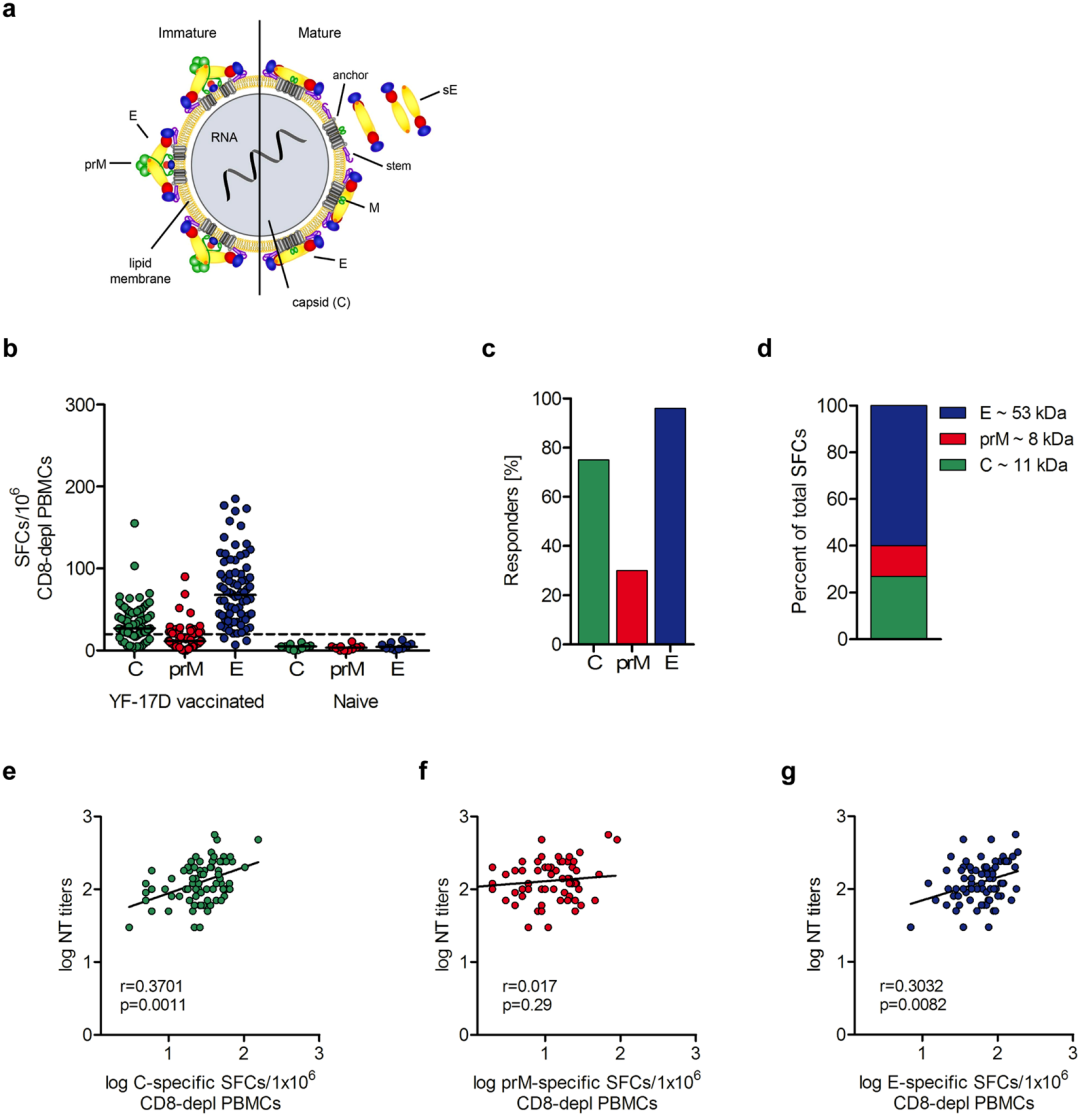
CD4 T cell and neutralizing antibody response to YF-17D vaccination. (**a**) Schematic representation of immature and mature flavivirus particles. The virion contains three structural proteins: C (capsid), prM (membrane) and E (envelope). The envelope of immature virions is covered by spikes of prM-E heterodimers. In mature virus particles, E proteins are arranged into homodimers. The soluble form of E (sE) lacks the membrane anchor and stem region. Reproduced from Vratskikh, *et al*.^68^. (**b**) Individual CD4 T cell responses to YF virus C, prM and E from 76 YF-vaccinated and 10 YF-naïve individuals as determined in IL-2 ELISPOT assays. Results are given as spot forming cells (SFCs). Medians are depicted as black lines. The dashed line represents the cut-off for assay positivity. (**c**) Responder frequencies to C, prM and E in 76 YF vacinees. (**d**) Percentage of spots contributed by C, prM and E peptides in 76 YF-17D vaccinees. (**e**–**g**) Spearman correlation of C- prM- and E-specific CD4 T cell responses and neutralizing Ab titers. Linear regressions are indicated by black lines.

Flavivirus particles consist of a nucleocapsid composed of multiple copies of the capsid protein C that contain the single-stranded, positive-sense RNA genome. The nucleocapsid is surrounded by a lipid envelope with two transmembrane proteins (E and prM) in immature particles (Fig. 1a, left panel)^23^. Virion maturation occurs in the trans Golgi network and is associated with a major rearrangement of E proteins at the particle surface that allows the proteolytic cleavage of prM into pr and M^24^, and leads to the formation of infectious virus particles (Fig. 1a, right panel). In mature virions, the E protein displays a herringbone-like arrangement of 90 dimers that cover the viral surface. Upon virus entry into host cells via receptor-mediated endocytosis, the acidic pH in the endosome triggers a structural reorganization of E from the metastable prefusion dimers into more stable postfusion homotrimers, driving the fusion of viral and endosomal membranes^25^.

Because of its important functions in virus entry, the E protein is the major target of neutralizing Abs, which mediate long-term protective immunity against flaviviruses^26^. E-specific B cells can receive help from T cells not only through the presentation of peptides derived from the E protein itself, but also from the two other structural proteins that are co-internalized (as part of the virus particle) by the E-specific B cell. Such intra-particle help has been shown for influenza^27, 28^ and hepatitis B viruses^29^ and was recently identified as a key mechanism in the Ab response against HIV^30^. In our study, we were interested in analyzing CD4 T cell help in the induction of E-specific neutralizing Abs and therefore studied the CD4 T cell specificities in YF vaccinated persons with peptides derived from all three structural proteins.

A subset of CD4 T cells, T follicular helper (Tfh) cells are particularly important for Ab production^31^. They express the chemokine receptor CXCR 5 that guides their migration into CXCL13-rich follicular regions in lymph nodes, where they support affinity maturation of B cells and differentiation into plasma cells^31^. Tfh cells reside within the lymphoid follicles, but a but a memory subset of Tfh cells circulates in the peripheral blood and can be identified by CXCR5 expression on memory (CD45RA^−^) CD4 T cells^32, 33^. In studies on seasonal influenza vaccines, the increase of blood memory Tfh cells which can be detected at day 7 after vaccination was found to correlate with the generation of Ab responses against influenza^34^. Their formation requires at least two steps, i) priming of CD4 T cells by pMHC II complexes presented on DCs and ii) sustained contact of primed CD4 T cells with B cells that present the same peptide to the TCR. Because of their outstanding role for Ab production, we analyzed the specificities of memory Tfh cells separately in addition to the bulk CD4 T cell responses.

We demonstrate that immunodominant CD4 T cell epitopes cluster within exposed strands and loops of E and in hotspots of two predicted helices of C. The immunodominance patterns generated in response to YF vaccination are very similar to those found after infection or vaccination with the distantly related TBE virus, suggesting a strong impact of structural protein features on the selection of epitopes in flavivirus CD4 T cell responses. In agreement with the results obtained for the bulk CD4 T cell response, all three structural proteins contributed to the memory Tfh cell response, but Tfh cells specific for the envelope proteins were overrepresented relative to non-Tfh cells.

## Results

### CD4 T cell responses to peptides of YF viral structural proteins C, prM and E

To determine the overall extent of CD4 T cell responses to YF viral structural proteins, peripheral blood mononuclear cells (PBMCs) obtained from 76 individuals at a median of 25 days (14–54 days) after YF vaccination and 10 YF-naive controls were depleted of CD8 cells and analyzed in IL-2 ELISPOT assays using pools of overlapping peptides spanning the entire sequences of YF virus C, prM and E (Fig. 1a). The results shown in Fig. 1b revealed responses against peptides of all three structural proteins with extensive individual variation. Responses to C, prM and E were detected in 75%, 30% and 96% of YF-vaccinated indiviudals, respectively (Fig. 1c). The number of days between YF vaccination and blood collection had no effect on individual ELISPOT results, as revealed by correlation analyses (Supplementary Fig. S2). On the average, 60.3% of the total CD4 T cell response was directed to E peptides, 27.3% to C peptides and 12.4% to prM peptides (Fig. 1d). Relative to their molecular weight, the response to C was twofold higher than the response to E. The results resemble the observations made with TBE virus for which a two- to threefold higher molar content of C compared to E was determined in purified virus particles^12^. YF-naïve individuals did not show a response to any of the peptides tested, confirming the specificity of the assay (Fig. 1b). The blood samples from all YF vaccinees were also analyzed for the presence of YF neutralizing Abs. As can be seen in Fig. 1e–g, there was a positive correlation between Ab titers and the extents of both, the C- and E-specific CD4 T cell responses. For prM, overall reactivities were very low and no statistical significant correlation was observed.

**Figure 2:**
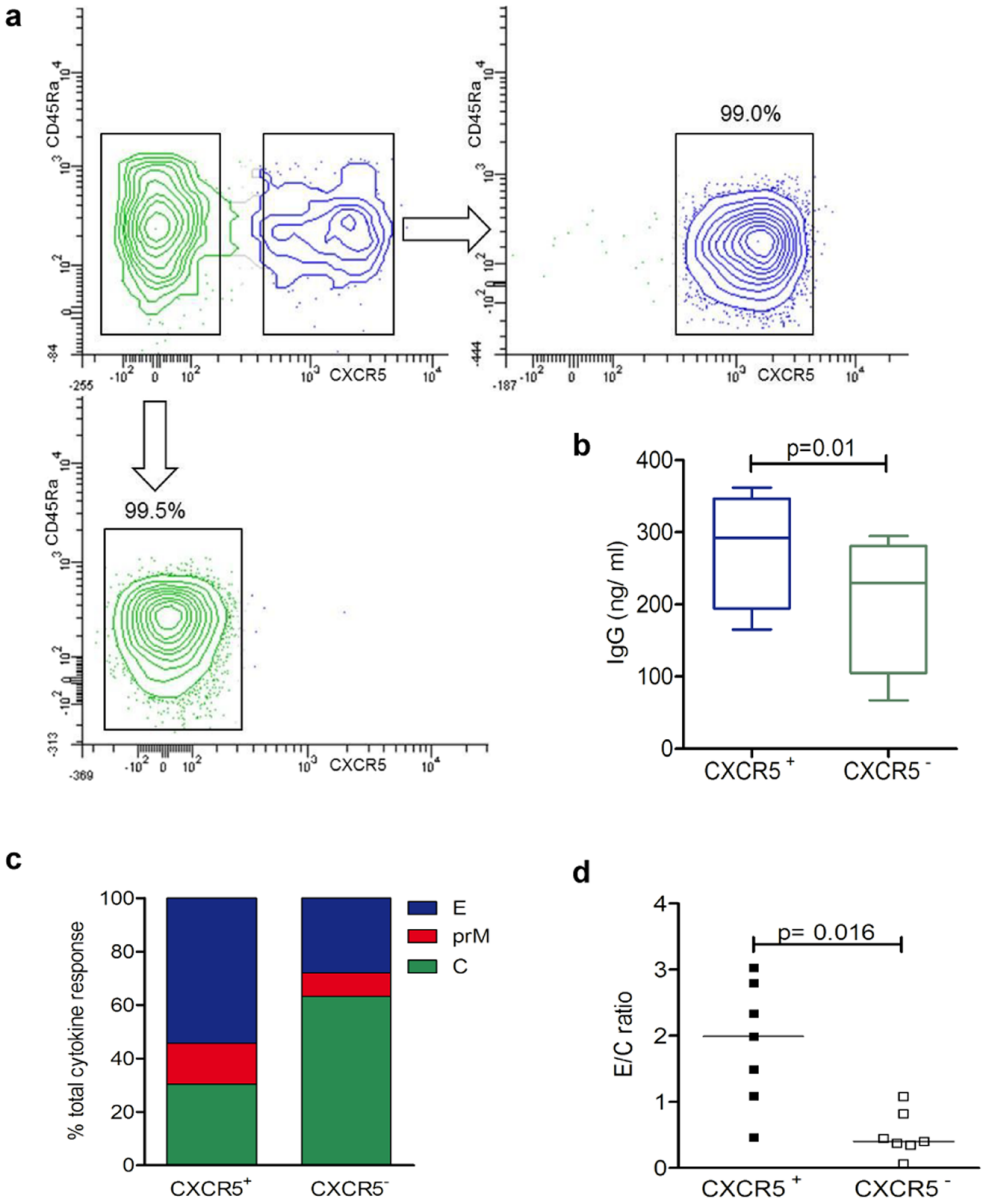
CXCR5^+^ and CXCR5^−^ CD4 T cell response to YF virus C, prM and E peptides. (**a**) CD4^+^ CD45Ra^−^ memory T cells from PBMC of healthy donors were sorted into CXCR5^+^ (upper right) and CXCR5^−^ cells (lower left). (**b**) IgG concentration revealed by ELISA in supernatants of sorted autologous B cells cultured for 10 days with either CXCR5^+^ or CXCR5^−^ cells in 4 independent experiments. Statistical signifiance was determined with the two-way ANOVA. (**c**) Percentage of cytokine events in sorted CXCR5^+^ and CXCR5^−^ subsets contributed by C, prM and E peptides as determined by intracellular cytokine staining. (**d**) Ratios of results with E and C peptides obtained in individual donors. Statistical signifiance was determined with the Wilcoxon matched-pairs signed rank test.

We next addressed the question whether the specificities of memory Tfh cells differed from those of bulk CD4 T cells. PBMCs were enriched for CD4 T cells and sorted into memory Tfh and non-Tfh effector cells using CXCR5 as a marker (Fig. 2a). In agreement with previous reports^32^, CXCR5 was expressed by 23.9% ± 7.9% and 22.9% ± 5.0% of memory (CD45RA^−^) CD4 T cells (mean ± SD) in YF-vaccinated and YF naïve individuals, respectively. We first determined the ability of the sorted T cell subsets to induce IgG production in cocultured autologous B cells. Quantitative measurement of IgG in cell culture supernatants after 10 days using IgG ELISA assays revealed that, in agreement with previous reports^35, 36^, CXCR5^+^ (memory Tfh) cells were more effective in providing help to Ab production, yielding higher IgG concentrations compared to their CXCR5^−^ (non-Tfh) counterparts (p = 0.01, two-way ANOVA) (Fig. 2b). Protein specificities of sorted subsets were compared using intracellular cytokine staining (ICS) after 10-day *in vitro* expansion with pools of C, prM and E peptides in the presence of autologous CD4-depleted PBMCs. The rationale for using ICS of short-term *in vitro* expanded cells was based on previous studies which showed that antigen-specific expansion of memory T cells improved the sensitivity for the detection of antigen-specific cells and on reports which indicated that ICS allowed the processing of much larger cell numbers than ELISPOT assays^37, 38^. Because there can be substantial heterogeneity in cytokine production after *in vitro* stimulation of CXCR5^+^ and CXCR5^−^CD4 T cells^33, 39^, the protein-specific CD4 T cells were determined based on IL-2, TNF-α and IFN-γ expression (see Supplementary Fig. S2). Figure 2c shows the proportions of cytokine responses to peptides of each of the three proteins obtained with CXCR5^+^ and CXCR5^−^ subsets. As can be seen in the figure, all three structural proteins contributed to the memory Tfh cell response similar to what was observed for bulk CD4 T cell responses. The direct comparison with non-Tfh cells in this assay, however, revealed different proportions of cells specific for these proteins with significantly lower ratios of E/C responses in non-Tfh compared to memory Tfh cells (p = 0.016, Wilcoxon matched-pairs signed rank test) (Fig. 2d). Together, these data demonstrate that capsid and envelope proteins significantly contribute to both, memory Tfh and non-Tfh effector cell responses, but the relative extent of C responses was higher in non-Tfh cells.

### CD4 T cell specificity and immunodominance

**Figure 3:**
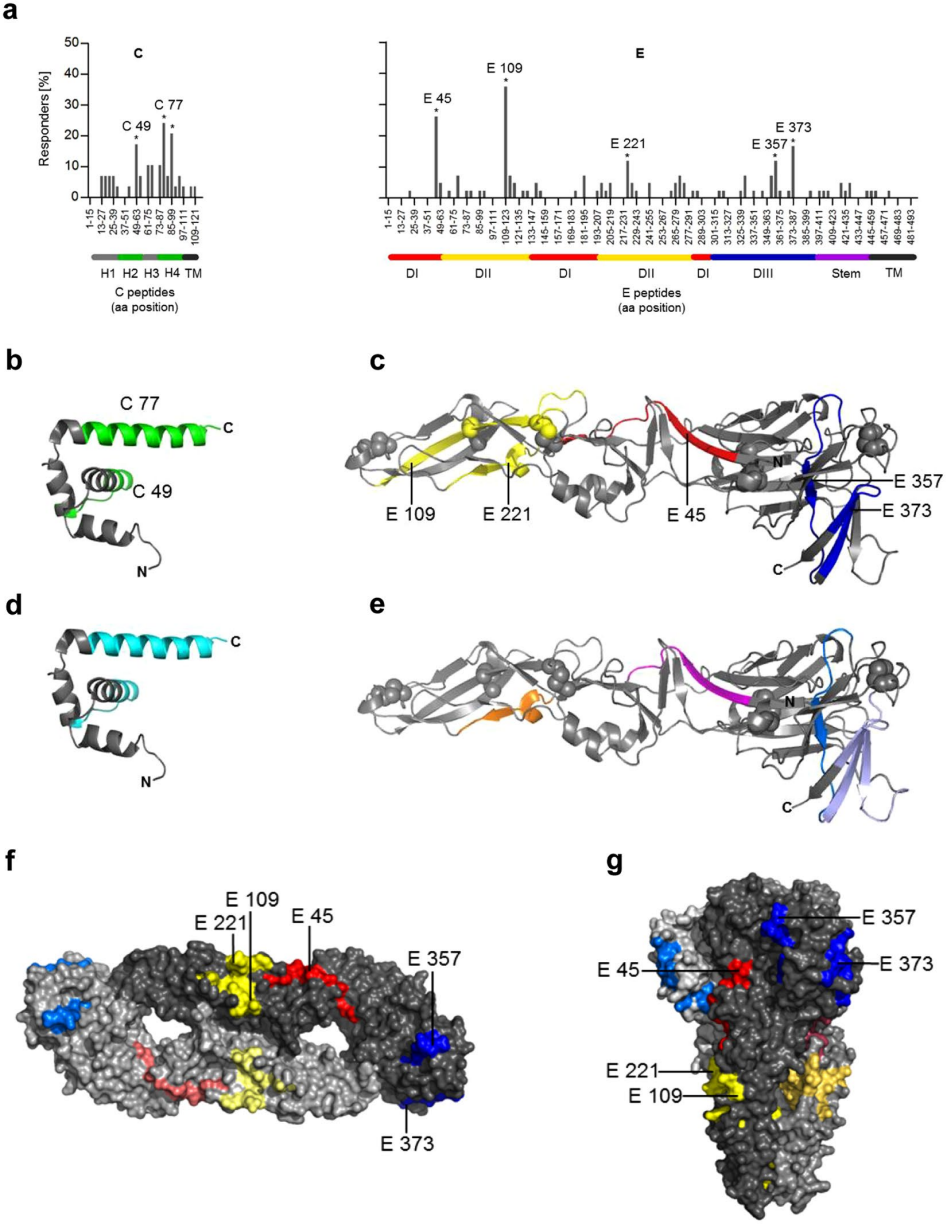
Mapping of immunodominant CD4 T cell responses specific for YF virus C and E proteins. (**a**) Percentage of positively tested YF-vaccinated persons recognizing a specific peptide within C (n = 29) and E (n = 42). Amino acid positions of peptides within the protein sequence are indicated on the x-axis. Peptides recognized significantly more often than the average are indicated by asterisks and are denoted by the first amino acid of the 15mer peptide used for single peptide testing (Fisher´s exact or chi-square test; p < 0.05). (**b,d**) Ribbon diagram of the crystal structure of the flavivirus Kunjin (KUN) C protein (PDB 1SFK)^40^, consisting of four helices (H1 to H4); for the amino-terminal region (grey line), no crystallographic data exist. (**c,e**) Ribbon diagram of the crystal structure of the TBE virus sE (PDB 1SVB)^16^ consisting of three domains (DI-III). (**b,c**) The dominant YF virus epitopes are colored as follows: C-green; E: domain I-red; domain II-yellow; domain III-blue. Spheres represent disulfide bridges. (**d,e**) Immunodominant epitopes derived from YF-17D and TBE cohorts identified at congruent positions are colored as follows: C protein (cyan); E protein DI (magenta), DII (orange) and DIII (blue); (**f,g**) surface representations of the neutral pH, dimeric form of sE in a top view and the postfusion trimeric form in a side view (PDB 1URZ)^22^. The immunodominant peptides are colored in each monomer according to corresponding domains: DI (red), DII (yellow) and DIII (blue).

To assess the epitope specificities of the CD4 T cell response to YF viral structural proteins, we performed ELISPOT analyses with peptide matrix pools as well as single peptides (Materials and Methods). The peptide specificities varied considerably between individuals (three representative examples are shown in Supplementary Fig. S3). To obtain information on immunodominance patterns, we calculated the frequencies of responses to single peptides using the cumulative data obtained from all individuals. These frequencies are illustrated in Fig. 3a as the percentage of responders out of all individuals recognizing at least one peptide of C (left panel) or E (right panel). Peptides C49 and C77 in C as well as E45, E109, E221, E357 and E373 in E were targeted significantly more frequently than expected under the null hypothesis of equal targeting frequencies for all peptides and were therefore defined as immunodominant epitopes (Fig. 3a). The same results were obtained when the evaluation was not based on the frequency but on the magnitude of responses (see Supplementary Fig. S4). Statistical analysis of prM epitope specificities could not be performed because of the low overall prM reactivity (Fig. 1b).

### Structural analysis of dominant epitope regions

High resolution structures of E have been determined for several flaviviruses^13–21^, whereas only a single flavivirus C protein structure (from WN/Kunjin virus^40^) is available. No such structures are yet available for YF virus. Under the assumption that the overall conformation of the YF virus structural proteins will be similar, we identifed the location of immunodominant YF CD4 T cell epitopes in the structures of Kunjin virus C and TBE virus E (Fig. 3b,c).

As can be seen in Fig. 3b, the response to C was strongly focused to helices H2 and H4, corresponding to predicted helices in the YF virus C protein (Materials and Methods). Peptides in helices H1 and H3 and in the N-terminal region of C (not resolved in the Kunjin C structure^40^) had much lower representation or did not elicit any response. In E, CD4 T cell epitope clusters were found in each of the three domains (one in domain I (E45), two in domain II (E109, E221) and two in domain III (E357, E373)) (Fig. 3c).

We then compared these newly identified YF virus epitopes with those previously determined in TBE patients and TBE-vaccinated individuals^12^. As can be seen in Fig. 3d, these comparisons revealed that in C, both immunodominant regions were at homologous positions in helices 2 and 4 (C49, C77). In E, 3 of the 5 dominant YF virus epitopes (E45, E221 and E357) were located at positions of dominant TBE virus epitopes (Fig. 3e). One YF virus epitope (E373) was located adjacent to sequence elements harboring a dominant TBE virus epitope. In one single case, a dominant YF virus epitope (YF E109) was not represented in TBE virus.

### Sequence conservation is not responsible for shared flavivirus epitope regions

We next analyzed whether the observed epitope overlap between YF and TBE viruses was due to sequence conservation in these regions. For this purpose, we calculated the percentage of identical amino acids in the aligned YF and TBE virus protein sequences for each dominant peptide region (Table 1). The overall sequence identities of the full-length E and C proteins were 42.7% and 30.1%, respectively. The matching epitope regions did not appear to be located at specifically conserved sites and showed sequence identities of 26.3% (E45), 38.1% (E221), 34.6% (E357), 14.3% (C49) and 45.2% (C77). The single YF epitope, however, which was not represented in the TBE cohorts mapped to a conserved sequence element in domain II (E 109, 67% sequence identity).

**Table 1:** Sequence identity between YF and TBE virus C and E proteins and congruent dominant epitope regions.

Protein	% identical amino acids	Peptide position (aa)	Amino acid sequence	% identical amino acids
C	30.1	TBE 37-57	LVLMRMMGILWHAVAGTARNP	14.3
		YF 43-63	GVQGFIFFFLFNILTGKKITA	
		TBE 65-95	SVPLKQATAALRKIKRTVSALMVGLQKRGKR	45.2
		YF 71-101	MLDPRQGLAVLRKVKRVVASLMRGLSSRKRR	
E	42.7	TBE 41-59	MDVWLDAIYQENPAKTREY	26.3
		YF 41-59	LDISLETVAIDRPAEVRKV	
		TBE 231-251	GAQN–WNNAERLVEFGAPHAVK	38.1
		YF 221-241	SGGV–WREMHHLVEFEPPHAAT	
		TBE 353-378	NVAMLITPNPTIENN–GGGFIEMQLPP	34.6
		YF 345-371	NKGILVTVNPIASTND–DEVLIEVNPPF	

### Surface exposure of immunodominant epitopes

It has been hypothesized that an epitope must be readily accessible to the MHC II molecule and/or proteolytic cleavage to become immunodominant^7^. As shown in protein surface representations, all three immunodominant YF virus epitope regions are located in exposed strands at the surface of the prefusion E dimer (Fig. 3f). At the acidic pH in the endolysosomal compartment of the APC, the E protein undergoes an irreversible structural change from a metastable dimer into a stable postfusion trimer. As shown in Fig. 3g, the immunodominant CD4 T cell epitopes are also accessible at the exposed surface of the low pH postfusion trimer^41^. Given that this conformational change can already occur at an early endosomal pH, it is possible that the postfusion trimer is an important source of T cell epitopes that provide help to B cells. Together, the data suggest that the epitope sites shared by YF and TBE viruses resulted from conserved structural features that favor their selection during antigen processing rather than from amino acid sequence conservation.

### *In silico* epitope prediction

**Figure 4:**
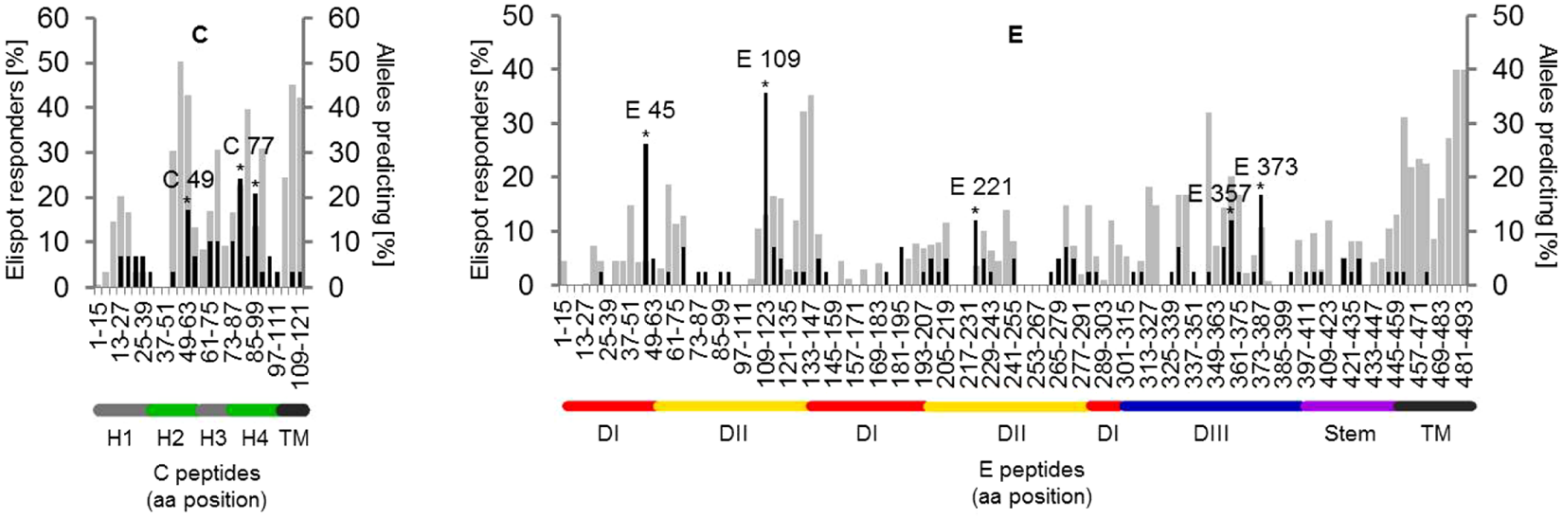
Comparison between experimentally identified CD4 T cell epitopes and *in silico* epitope predictions based on peptide-MHC II affinity. Percentages of HLA class II alleles predicting a specific 15-mer peptide for C (left panel) and E (right panel). The experimentally defined immunodominant epitopes are denoted by the first amino acid of the 15mer peptide used for single peptide testing; *in silico* predicted epitopes (grey), experimentally determined epitopes (black).

Epitope prediction based on peptide-MHC II affinity was performed for each peptide tested using the IEDB database. For this purpose, the HLA class II alleles (HLA II -DRB1, -DRB3/4/5, -DP and -DQ) were determined. The entire list of 412 HLA class II alleles determined in 76 YF vaccinated individuals can be found as Supplementary Table S1. For each peptide and 62% of the different HLA class II alleles, the IEDB provided a percentile rank score. For comparison of experimental and predicted data, we used a population-based approach in which all alleles with a percentile rank score of ≤5 were pooled into a prediction map. The frequency of binding alleles was calculated by dividing the number of alleles with a percentile rank score ≤5 for each peptide by the number of alleles present in the cohort. This frequency is illustrated in Fig. 4 as the percentage of binders out of all alleles.

Overall, 89.5% (17/19) and 90.7% (49/54) of the identified peptides in C and E, respectively, had an IEDB percentile rank of 5 or lower. For C, the immunodominant areas were also among the most frequently predicted (Fig. 4). In the E protein, the immunodominant epitopes were not frequently predicted as top binders and peptides frequently predicted with high MHC II affinities, especially from the transmembrane domains, were clearly underrepresented in the CD4 T cell response (Fig. 4). In an analysis of the percentages of predicted peptides that were also identified experimentally, we found that for the C protein 70% (17/24) of the predicted peptides yielded ELISPOT responses, whereas in the E protein, such a concordance was obtained in only 46.6% (41/88).

## Discussion

The goal of this study was to gain insight into the specificities and immunodominance patterns of CD4 T cells that can provide direct help to B cells for the production of neutralizing Ab after YF vaccination. This was accomplished by determining the repertoire of epitope specificities in response to the viral structural proteins and correlating immunodominance patterns with structural features of these proteins as well as *in silico* predicted peptide-binding affinities. In addition, the newly identified YF epitopes were compared to those obtained with the distantly related flavivirus TBE virus to investigate the role of protein structural features on immunodominance. Flaviviruses are an excellent model system to address these issues, because their proteins are structurally homologous but highly diverse in amino acid sequence.

The study focused on responses to epitopes within the viral structural proteins, all of which are internalized as part of the virus particle by E-specific B cells and thus can provide direct help to B cells for neutralizing Ab production. The CD4 T cell response is of critical importance for protective immunity against YF^5^, and within each protein, only few epitopes were identified as immunodominant. So far, the mechanisms responsible for the generation of this immunodominance are still poorly understood. Previous studies suggest that in addition to host specific factors, protein structural features, which influence epitope processing may play a role. Our study corroborates such a structural influence by demonstrating that the response was strongly focused to the predicted helices H2 and H4 of C and to exposed strands and loops of the three E protein domains. Other studies with DEN, JE, WN and YF have also identified CD4 T cell responses toward peptides in these regions^42–46^.

Further evidence for the contribution of protein structural features on epitope dominance was obtained by a comparison of YF virus epitopes with those identified in TBE virus^12^. Considering that the overall sequence identities of full-length C and E proteins of these viruses are only 30% and 42%, respectively, it was remarkable that 5 out of the 7 immunodominant YF virus epitopes were found to be located at positions homologous to those in TBE virus. Specifically, the peptide regions in helices 2 and 4 of the C protein, as well as three out of the five epitopes in E (E45, E221 and E357) were virtually superimposable, and the YF virus epitope E373 mapped to a sequence adjacent to a dominant TBE virus epitope. The single immunodominant YF virus epitope, not represented in TBE responses (E109), was located in sequence elements of domain II that are constrained by two disulfide bonds. The processing of this epitope could depend on the activity of GILT (γ-interferon-inducible thiol reductase) which mediates reduction of disulfide bonds in the lysosomes of APCs^47, 48^. It has been shown that TBE but not YF virus is able to antagonize IFN-γ-induced gene expression via STAT1 inhibition^49^. The dominance of epitope E109 in YF virus but not in TBE virus could therefore result from a difference in the specific viral antagonism of the host immune response.

Shared epitope regions in YF and TBE viruses could be due to amino acid sequence conservation. In our comparison between YF and TBE viruses, however, this was not the case because amino acid sequences of the shared epitope regions in E differed strongly between YF and TBE viruses and displayed only 26–38% amino acid identity. Together, the data suggest that the similarity of immunodominant regions in TBE and YF viruses are due to conserved structural features of flavivirus proteins that favor their selection during antigen processing, rather than amino acid sequence conservation.

The congruencies of experimental and *in silico* predicted data differed substantially between specific regions in C and E. Although the predicted C-terminal peptides in the capsid protein did not induce an immunodominant CD4 T cell response, we found that the dominant epitope regions in helices 2 and 4 were also among the most frequently predicted. For E, some experimentally determined epitopes (E45, E109 and E221) had low predicted MHC II affinities and others, especially from the transmembrane domain, that were among the most frequently predicted, were clearly underrepresented in the CD4 T cell response. Such peptides may not be available for processing, because they are shielded in lipid membranes or constrained by other structural features not considered in MHC affinity prediction. The results corroborate previous analyses involving a large set of viral envelope proteins from different viruses^12, 50, 51^ and suggest that three-dimensional structure plays an important role in guiding epitope presentation from these proteins. In line with these findings, new prediction algorithms that address such structural constraints of antigen processing have recently been proposed to facilitate accurate epitope prediction^52^. Alternatively, differences in the frequency of naïve T cells specific for individual epitopes could have an influence on CD4 T cell immunodominance^53^. This may particularly apply to peptides homologous to self pMHC ligands, as T cells that interact too strongly with such peptides are deleted from the naïve T cell repertoire. Since the IEDB predictive algorithms are limited to the identification of peptides with high MHC binding affinities, the discrepancies observed between high predictive values and experimentally determined immunodominance hierarchies may be associated with a poor generation of such peptides during antigen processing or deficiencies in the T cell repertoire due to tolerance mechanisms.

Our findings are consistent with the notion that accessibility to proteolytic processing and/or MHC II binding is an important feature that determines immunodominance. Evidence for this also comes from studies of influenza and HIV proteins which showed that CD4 T cell epitopes cluster at exposed protein surfaces or at flanks of conformationally flexible protein segments^8–11^. Such segments may serve as entry points for initial proteolytic processing and are therefore more easily unfolded to expose the epitopes for binding to MHC II molecules^52, 54^. In the case of flaviviruses, the E protein undergoes an acidic pH-triggered structural change after receptor-mediated endocytosis, from a metastable dimer into a stable postfusion trimer^41^. Mapping of the immunodominant peptides on the crystal structure of the postfusion trimer revealed that all were accessible at the exposed surface of the molecule. Given that fusion can already occur at an early endosomal pH, the trimeric postfusion conformation is likely a substrate for proteolytic processing and could serve as a source of helper T cell epitopes. For the C protein, links of immunodominance and surface exposure are difficult to establish, because the structure of the RNA-capsid protein complex, i.e. the viral core, is yet unresolved and it is unknown how processing proceeds after the core has been released during the process of viral membrane fusion.

It was an interesting finding of our study that the memory response of Tfh cells which have the potential to support Ab production differed from that of non-Tfh effector cells. Specifically, the non-Tfh cell response to C was substantially higher than that to E and prM. The role of this subset is unclear, but one could speculate that the C-specific non-Tfh response contributes to other CD4 T cell-mediated effector mechanisms, such as provision of key antiviral cytokines or direct cytotoxicity, as previously shown for dengue virus^55^. Since our *ex vivo* ELISPOT assays measured only IL-2, future investigations will need to assess the functional relevance of responses directed towards the identified immunodominant epitopes. Consistent with our findings, recent studies with HIV and influenza virus, reported distinct antigen specifities of Tfh and non-Tfh cells^35, 56^. The major difference between the two CD4 T cell subsets is that memory Tfh cells, unlike non-Th effector cells, require a second step of cognate interaction with antigen-specific B cells for full lineage commitment^31^, and therefore, differences in antigen presentation by APC subtypes could play a role. In particular, overrepresentation of E and prM in Tfh cell responses would mean that B cells presented proportionally higher amounts of peptides from envelope proteins at the expense of C. This may be explained by the fact that processing and presentation by B cells is substantially more efficient when the antigen is bound to the BCR^57, 58^. In the case of BCR-mediated uptake of virions, epitopes from surface-exposed, Ab-accessible envelope proteins would be favored in comparison to the internally located capsid. Also, differences in endosomal acidification that triggers viral fusion and the subsequent delivery of nucleocapsids into the APC cytoplasm can lead to different processing pathways and explain the proportionally higher response to E peptides in memory Tfh cells. More detailed analyses to determine precisely whether pre- and/or post-fusion conformations of the E protein serve as a source for the peptides presented by different types of APC will be needed to clarify this point.

In conclusion, we identified a strong CD4 T cell response to epitopes derived from the C and E proteins of YF virus. The restriction of immunodominant epitopes to exposed E protein surfaces and their similar positioning within proteins of distantly related flaviviruses is consistent with a strong influence of protein structural features that shape CD4 T cell epitope selection and provide leads for a rational design of immunogens for vaccination and for analyzing T cell responses of vaccine candidates.

## Materials and Methods

### Study design

The study cohort consisted of 76 subjects (42 f, 34 m; age range, 18–82 years) from whom PBMC samples were obtained 14–54 days (median, 25 days) after vaccination with the YF virus strain 17D-204 (STAMARIL®; Sanofi Pasteur). Blood samples of ten healthy donors obtained at the Austrian Red Cross and the Department of Virology, Medical University of Vienna, were used for cell sorting experiments. In these samples, previous YF vaccination was confirmed by YF virus neutralization assays. As a control, samples of ten YF-seronegative individuals (6 f, 4 m; age range, 22–47 years) who had no history of YF vaccination or infection were analysed.

### Ethics statement

The ethics committee of the medical association Hamburg approved the study protocol, and all study participants provided written informed consent. Study procedures were carried out in accordance with the ethical standards of the Declaration of Helsinki.

### Preparation of blood samples

PBMCs were isolated from blood samples using Ficoll-Paque Plus™ (GE Healthcare) and cryopreserved in liquid nitrogen. Plasma samples were stored at −20 °C. For coculture experiments, B cells were enriched from whole blood samples using RosetteSep^TM^ B cell enrichment cocktail (STEMCELL Technologies, Grenoble, France), as described previously^59^.

### Depletion of CD8 cells

CD8-depletion was performed using anti-CD8 antibody-coupled magnetic beads and LD columns (Miltenyi Biotec GmbH, Germany), as previously described^12^. CD8-depleted PBMCs were resuspended in serum-free medium (AIM-V; Gibco), incubated overnight at 37 °C in 5% CO_2_, and resuspended at a final concentration of 2 × 10^6^ cells/ml in AIM-V for use in IL-2 ELISPOT assays. The viability and purity of CD8-depleted PBMCs in each sample were assessed by flow cytometry using anti-CD3- PE, anti-CD8-APC, anti-CD4-PacificBlue™ and 7-aminoactinomycin D (7-AAD) (all purchased from BD Bioscience). The CD8-depleted PBMC samples contained less than 1% CD8 T cells.

### Peptides

For T cell analysis, 188 peptides were purchased from JPT (Berlin, Germany). The 15mer peptides overlapping by 11 amino acids cover the entire sequences of C, prM and E from the virus 17D strain (Swiss-Prot P03314). The purity of peptides was >70%, as determined by high-performance liquid chromatography. Lyophilized peptides were dissolved in dimethyl sulfoxide and diluted in AIM-V medium. Peptides were arranged into three master pools covering the complete C, prM and E protein sequences. Matrix pools of C (n = 11), prM (n = 13) and E (n = 22) contained up to 11 peptides with each peptide present in two distinct pools. All positive matrix pool results were confirmed by testing the samples with single peptides in independent experiments.

### IL-2 ELISPOT assay

IL-2 ELISPOT assays were performed, as described previously^12^. Briefly, ELISPOT plates (Merck-Millipore) were treated with 70% ethanol for 30 minutes prior to coating with 1 µg anti-IL-2 antibody (3445-3-1000, Mabtech). The plates were blocked with RPMI 1640 medium (Sigma) containing 10% human serum, 1% penicillin/ streptomycin/glutamine (Gibco) and 1% nonessential amino acids (Sigma). 2 × 10^5^ CD8-depleted PBMC were added per well, and were stimulated with peptides at a final concentration of 2 µg of each peptide/ ml. AIM-V medium or phytohemagglutinin (PHA, Sigma) at a final concentration of 0.5 µg/ ml were used as controls. PBMCs were incubated for about 45 hours at 37 °C and 5% CO_2_. Plates were washed twice with PBS containing 0.05% Tween 20 and twice with PBS. Detection of IL-2 spots was performed with 0.05 µg biotin-conjugated anti-IL-2 antibody (3445-6-250, Mabtech), streptavidin-coupled alkaline phosphatase (ALP; 1:1000, 3310-10, Mabtech) and 5-bromo-4-chloro-3-indolylphosphate/nitroblue tetrazolium (BCIP/NBT; B5655, Sigma). The spots were analyzed using a Bio-Sys Bioreader 5000 Pro-S/BR177 and Bioreader software, generation 10. Data were calculated as spot forming cells (SFCs)/10^6^ CD8-depleted PBMCs after subtraction of the negative control (mean spot number from three to four unstimulated wells), as described previously^12^. A single peptide response was defined positive if the corresponding master pool, matrix pool as well as single peptide testing yielded >20 SFCs/10^6^ CD8-depleted PBMCs.

### Cell sorting of CXCR5^+^ and CXCR5^−^ T cells

CD4 cells were enriched using a CD4 T cell isolation kit, according to the manufacturer´s instructions (Miltenyi Biotec GmbH, Germany). The CD4 enriched cells were separated into two populations by flow cytometric sorting using anti-CD4-PacificBlue™, anti-CD45Ra-FITC and CXCR5-Alexa-Fluor 647 (all purchased from BD Bioscience). The resulting populations were routinely >98% pure.

### In vitro activation and coculture experiments

5 × 10^4^ sorted CD4^+^ CD45Ra^−^ CXCR5^+^ or CD4^+^ CD45Ra^−^ CXCR5^−^ T cells were cultured with 5 × 10^4^ autologous B cells in the presence of 1 µg/ml staphylococcal enterotoxin B (SEB) to facilitate cognate cell-cell interactions, as described previously^35^. Culture supernatants were harvested after 10 days and analyzed for IgG using a Human IgG ELISA Quantitation Set (Bethyl Laboratories, Montgomery, Texas), according to the manufacturer’s instructions. For comparison of protein specificity, sorted T cell subsets (1–2 × 10^5^/well) were restimulated for 10 days with peptide pools containing the entire C, prM or E sequence (1 µg/ml) in the presence of autologous PBMCs depleted of CD4 cells. IL-2 (50U/ml) was added at days 0 and 6.

### Intracellular cytokine staining and flow cytometry

Intracellular cytokine staining was performed as described previously^60^. The staining cocktail included anti-IFN-γ-FITC, anti-TNF-α-PE-Cy7, anti-IL-2-APC, anti-CD3-APC-H7, anti-CD4-Pacific Blue and ViViD-AmCyan. All mAb reagents were obtained from BD Bioscience. Cells were analysed on a FACS CantoTM II (BD, USA) cytometer. Data were further analysed using BD Diva software version 6.1.2 or FlowJo software version 7.2.5 (Tree Star, USA)^61^. Cytokine-positive CD4 T cells were identified by sequential gating: Dead cells were excluded by gating on lymphocytes (FSC vs SSC plot) and on ViViD negative cells (see Supplementary Fig. S2). The lymphocyte gate included quiescent and activated lymphocytes, which display increased FSC and SSC signals, as reported previously^62, 63^. Subsequently, lymphocytes were gated on CD3 and CD4 T cells. All events were normalized to 1 × 10^5^ CD4 T cells. Boolean gating function of FlowJo was used to determine the frequency of each response based on all possible combinations of cytokines, except for single TNF-α subsets, which show high background staining, as reported previously^60, 64^. The gates for detection of cytokines in peptide-stimulated cell samples were set in the samples with no antigen stimulation. A lower threshold, corresponding to ≥twofold above the samples with no antigen stimulation, was built for each specific functional combination. Total frequencies of antigen-specific cells were calculated by summing the events within each unique combination of functions (counting each responding cell only once) (see Supplementary Fig. S2).

### Neutralization assays

TBE virus neutralization tests (NT) were carried out in microtiter plates using baby hamster kidney cells (ATCC BHK-21), as described previously^65^. Briefly, two-fold serial dilutions of heat-inactivated serum samples in duplicates were incubated with 50–100 TCID_50_ of virus for 1 h at 37 °C. BHK-21 cells were added, and incubation was continued for 3 days. The presence of virus in the supernatant was assessed by ELISA, and NT titer was defined as the reciprocal of the serum dilution that gave a 90% reduction in the absorbance readout in the assay (NT_90_) compared to the control without antibody. For YF virus NTs, heat-inactivated serum samples (starting at a dilution of 1:20) were incubated with 40–80 TCID_50_/well of YF virus for 1 h at 37 °C. The presence of virus in the supernatant was assessed by the occurrence of cytopathic effects. NT titers ≥20 were considered positive.

### Structural analysis

For epitope assignment onto the crystallographic structures of TBE virus E and KUN virus C proteins, multiple sequence alignment was performed for E protein (NCBI accession number: YF-17D, NCBI NP_740305.1; TBE Neudörfl, NCBI NP_775503.1; WN virus, NCBI AGX89731.1; Zika virus, NCBI AMD16557.1, DEN 2 virus, NCBI NP_739583.2 and JE virus, NCBI NP_775666.1) and C protein (YF-17D, NCBI NP_775998.1; TBE Neudörfl, NCBI NP_775499.1; WN virus, NCBI AGX89731.1; Zika virus, NCBI AMD16557.1; DEN 2 virus, NCBI NP_739591.2 and JE virus, NCBI NP_775662.1), using the PROMALS online tool (http://prodata.swmed.edu/promals/promals.php). Secondary structures of YF virus C protein were predicted by use of PSIPRED algorithm (http://bioinf.cs.ucl.ac.uk/psipred).

### Analysis of amino acid sequence homology

Protein sequences of YF (Swiss-Prot P03314), TBE (NCBI GI 27596775; GI 27596778), WN (NCBI AGX89731.1), DEN2 (NCBI NP_739591.2 and NP_739583.2), JE (NCBI NP_775662.1 and NP_775666.1) and Zika (NCBI AMD16557.1) viruses were aligned, using Clustal Omega software.

### HLA genotyping

Genotyping of HLA-DRB1, HLA-DQB1, and HLA-DPB1 alleles was performed by nucleotide sequencing of the whole gene, genotyping of HLA-DRB3/4/5 alleles from exon2 to the 3′UTR by next generation sequencing as described previously^66^. Briefly, long range PCR was performed with home-made primers, libraries generated by enzymatic fragmentation of the amplicons. After ligation to barcoded adapters the libraries were subjected to an emulsion PCR and the product sequenced on an Ion Torrent sequencing device (IonTorrent PGM, Life Technologies, Thermo Fisher Scientific Inc., Waltham, MA). Analysis of sequences was done with HLATypeStream (Thermo Fisher Scientific Inc., Waltham, MA) and NGS GenDX (GenDX, Utrecht, NL) software. HLA-types were assigned on basis of the IMGT/HLA database.

### MHC II epitope prediction

The MHC II epitope predictions were made on 09/08/2016 using the IEDB analysis resource Consensus tool, which achieved better overall performance than all existing individual MHC II prediction tools^61, 67^. Amino acid sequences of C and E proteins of YF virus (Swiss-Prot P03314) were entered separately. Epitope predictions were performed for all peptides that were used in IL-2 ELISPOT assays and the experimentally determined HLA II alleles that were available in the IEDB (Supplementary Table S1).

### Statistical analysis

Immunodominant peptides were defined using a chi-square test. Spearman-rho correlation coefficient was used to test for an association between CD4 T cell responses and NT titers. Two-way ANOVA was used for comparison of IgG values in culture supernatants. The statistical analysis of differences in E to C ratios between sorted T cell populations was done with the Wilcoxon matched-pairs signed rank test. Wilcoxon matched-pairs signed rank test is the best statistical test in this case because of the direct comparison of T cell subsets within donors. All P values were two-sided, and values < 0.05 were considered statistically significant. The software program Prism 5.0 (Graph Pad) was used for all statistical tests.

### Data availability

All data generated or analysed during this study are included in this published article (and its Supplementary Information files).

## Electronic supplementary material

Supplementary Information
