# Supplementary material for: Protein structure shapes immunodominance in the CD4 T cell response to yellow fever vaccination
Source: Sci Rep. 2017 Aug 21;7:8907. doi: 10.1038/s41598-017-09331-w (PMC5566484; doi:10.1038/s41598-017-09331-w)
Supplement: Supplementary file 1 — Supplementary Information [file 41598_2017_9331_MOESM1_ESM.pdf]

# Supplementary Results

# Protein structure shapes immunodominance in the CD4 T cell response to yellow fever vaccination

**Maximilian Koblischke, Maria S. Mackroth, Julia Schwaiger, Ingrid Fae, Gottfried Fischer, Karin Stiasny, Franz X. Heinz & Judith H. Aberle**

### Supplementary Table S1: HLA class II alleles of YF vaccinated individuals

| **DRB1** | **DRB3/4/5** | **DQB1** | **DPB1** |
|---|---|---|---|
| 01:01 (13)[a] | 3*01:01 (33) | 02:01 (13) | 01:01 (10) |
| 01:02 (1) | 3*02:02 (24) | *02:02[b] (6)* | 02:01 (19) |
| 03:01 (20) | 3*03:01 (1) | 03:01 (22) | *02:02 (1)* |
| 04:01 (18) | 4*01:01 (5) | 03:02 (17) | *03:01 (15)* |
| 04:02 (3) | 4*01:03 (29) | *03:03 (8)* | 04:01 (52) |
| 04:04 (4) | 5*01:01 (19) | *03:19 (1)* | 04:02 (13) |
| 04:08 (1) | 5*01:02 (1) | 04:02 (2) | 05:01 (2) |
| 07:01 (13) | 5*01:08 (1) | 05:01 (13) | *06:01 (2)* |
| 08:01 (2) | 5*02:02 (2) | *05:02 (10)* | *10:01 (2)* |
| 08:03 (2) | | *05:03 (1)* | *11:01 (1)* |
| 08:04 (1) | | *05:04 (1)* | *14:01 (2)* |
| 11:01 (14) | | *06:01 (2)* | *15:01 (2)* |
| 11:03 (3) | | 06:02 (20) | *16:01 (1)* |
| 11:04 (6) | | *06:03 (18)* | *17:01 (1)* |
| 12:01 (1) | | *06:04 (9)* | *19:01 (3)* |
| 13:01 (13) | | *06:09 (1)* | *20:01 (1)* |
| 13:02 (2) | | | *23:01 (2)* |
| 14:45 (1) | | | *85:01 (1)* |
| 15:01 (20) | | | *104:01 (1)* |
| 15:02 (1) | | | |
| 15:03 (1) | | | |
| 16:01 (4) | | | |
| 16:02 (1) | | | |

[a] the number of individuals carrying the allele is indicated in parenthesis
[b] for alleles indicated in italics no prediction algorithm was available

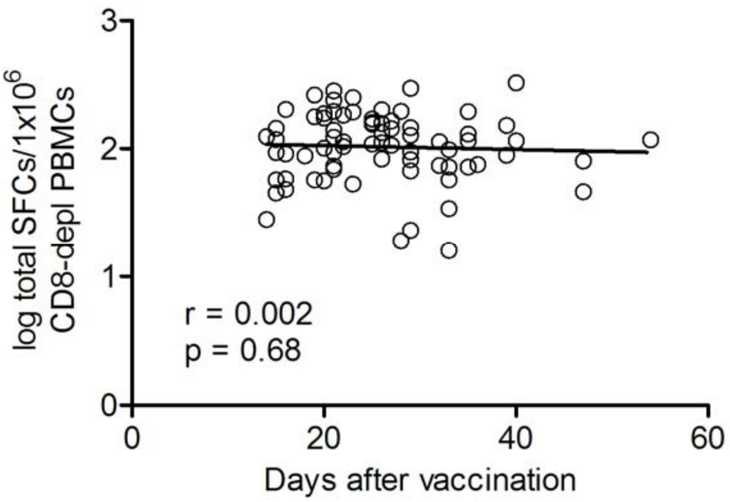


**Supplementary Figure S1. Correlation of total IL-2 ELISPOT responses after YF vaccination.** The Pearson correlation coefficient r and the p value are indicated. Linear regression is indicated by a black line. Results are given as spot forming cells (SFCs).

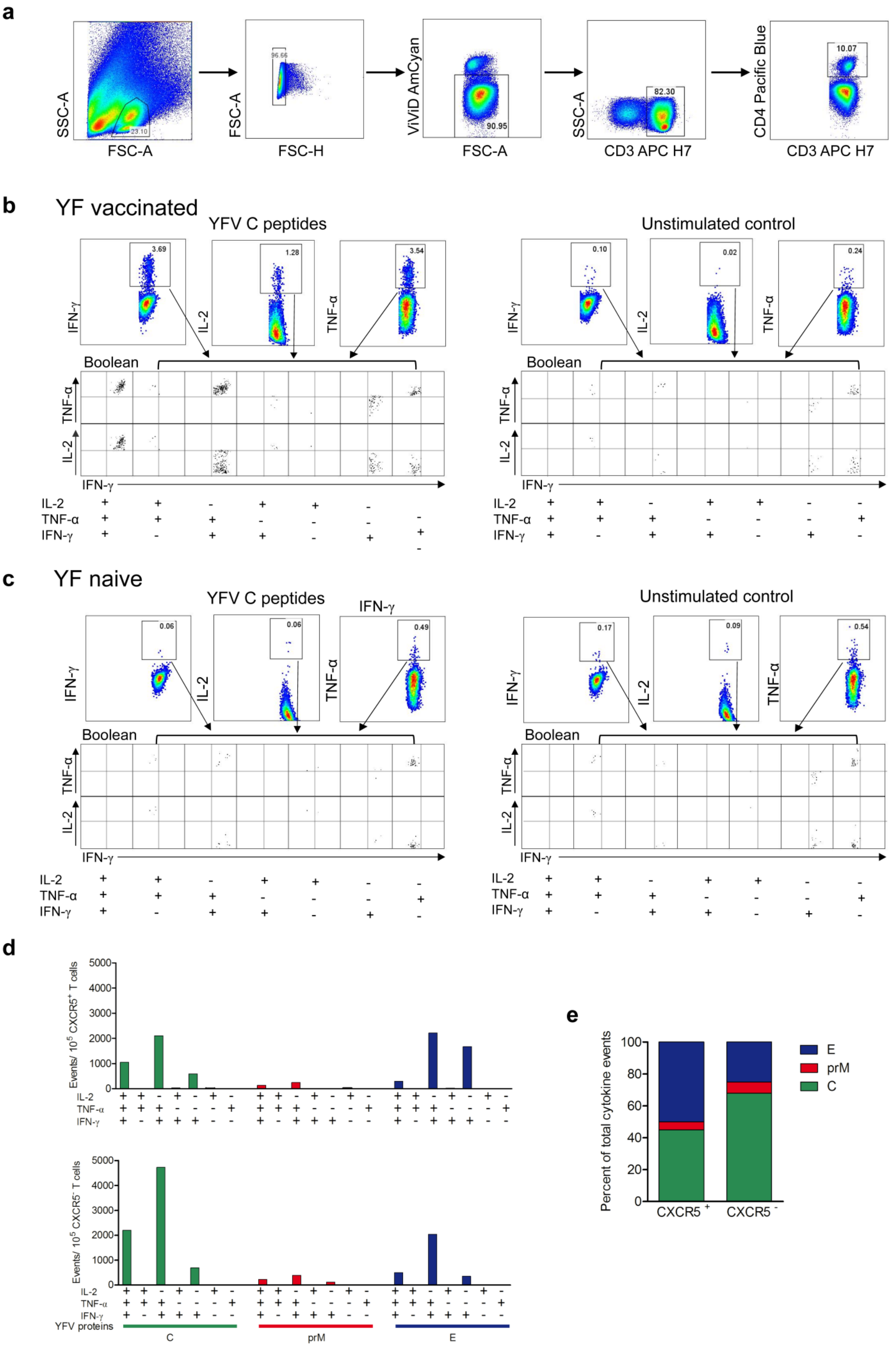
a
SSC-A
FSC-A
23.10
FSC-A
FSC-H
96.66
ViViD AmCyan
FSC-A
90.95
SSC-A
CD3 APC H7
82.30
CD4 Pacific Blue
CD3 APC H7
10.07
b
YF vaccinated
YFV C peptides
Unstimulated control
IFN-γ
IL-2
TNF-α
3.69
1.28
3.54
0.10
0.02
0.24
Boolean
IFN-γ
IL-2
TNF-α
c
YF naive
YFV C peptides
Unstimulated control
0.06
0.06
0.49
0.17
0.09
0.54
Boolean
d
Events/ 10^5 CXCR5+ T cells
Events/ 10^5 CXCR5- T cells
IL-2
TNF-α
IFN-γ
YFV proteins
C
prM
E
e
Percent of total cytokine events
CXCR5 +
CXCR5 -
E
prM
C

**Supplementary Figure S2. Measurement of YF virus-specific CD4 T cells in sorted CXCR5$^+$ and CXCR5$^-$ cells after in vitro stimulation with C, prM and E peptide pools. (a)** Representative gating of sorted CXCR5$^+$ CD45Ra$^-$ memory CD4 T cells after a 10-day coculture with autologous CD4-depleted PBMCs. The lymphocyte gate includes activated lymphocytes, which exhibit increased FSC and SSC signals relative to quiescent lymphocytes. **(b,c)** Representative FACS plots of intracellular cytokine staining and corresponding Boolean gate analyses of sorted CXCR5$^+$ memory CD4 T cells from a YF-vaccinated donor **(b)** or a YF naïve donor **(c)** after a 10-day coculture. CD4 T cells that produced IFN-γ, IL-2 and TNF-α or combinations of these cytokines were separately identified in CXCR5$^+$ and CXCR5$^-$ subsets. The gates for detection of cytokines in peptide-stimulated cell samples were set in the samples with no antigen stimulation (unstimulated control). Boolean gate analyses were applied to determine the frequencies of the seven non-overlapping cytokine subsets, each representing one of the possible cytokine combinations. **(d)** Column representation of the same YF vaccinated subject´s CD4 T cell response after Boolean gating. The three cytokines in each of their seven possible combinations are indicated on the x-axis. Columns represent total cytokine events/ $10^5$ CD4 T cells in CXCR5$^+$ (top) and CXCR5$^-$ (bottom) populations. The responses to C, prM and E peptide pools are shown in green, red and blue, respectively. Total virus-specific CD4 T cell responses were calculated by summing the events within each combination of cytokines. (**e**) Percentage of total events contributed by C, prM and E in CXCR5$^+$ and CXCR5$^-$ populations from the representative YF vaccinated subject.

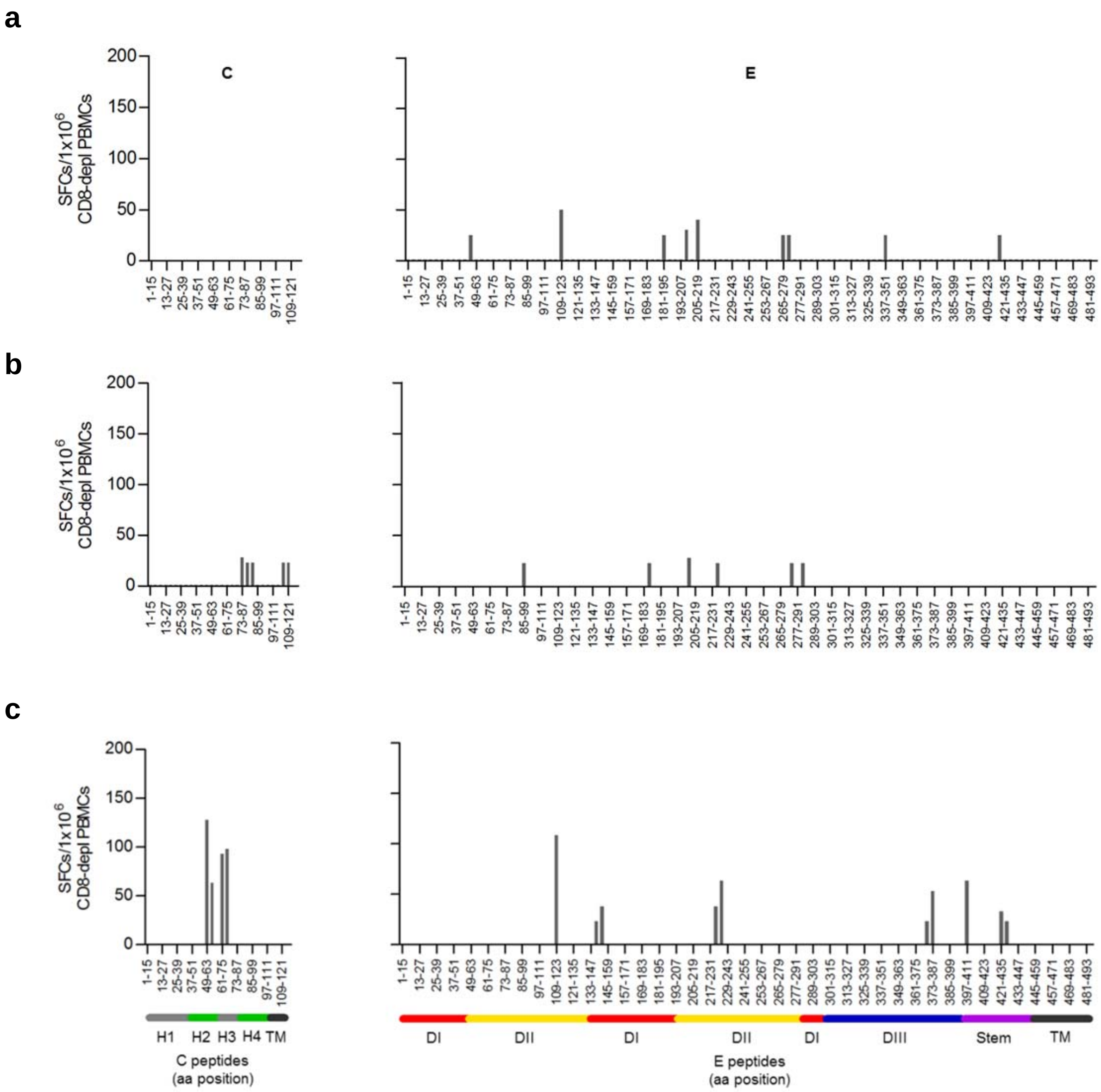


**Supplementary Figure S3. Individual CD4 T cell peptide specificities to YF virus C and E proteins.** (**a-c**) Representative examples of three YF-17D vaccinated subjects, indicating CD4 T cell responses against 15mer peptides derived from YF virus C and E proteins determined using IL-2 ELISPOT assays. Amino acid positions of peptides refer to their location in the protein sequence of C and E, respectively, starting at the N-terminus of each protein. Colored lines below the x-axes indicate positions of α-helices one to four (green, grey) and the transmembrane domain (TM, black) of the C protein, as well as E protein domains DI (red), DII (yellow) and DIII (blue), stem (purple) and TM (black).

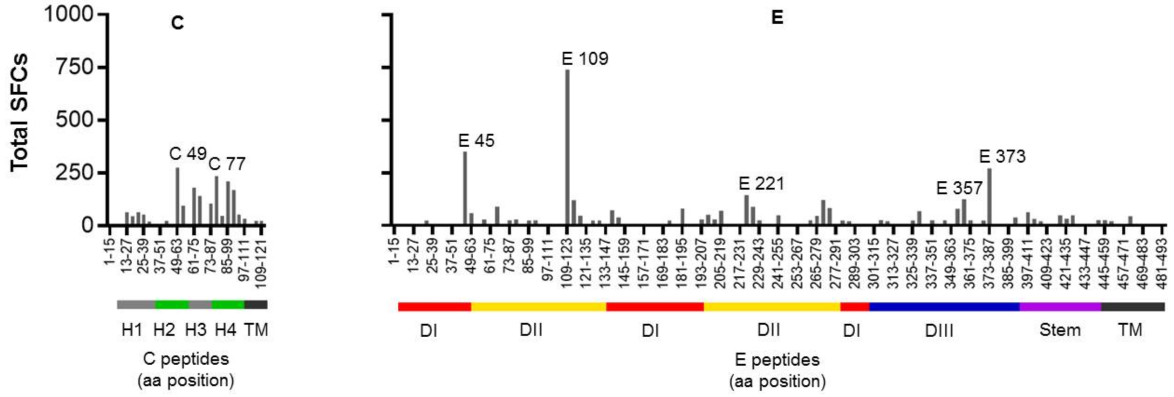


**Supplementary Figure S4. Magnitude of CD4 T cell responses to single peptides within the YF virus C and E proteins.** Total numbers of IL-2 spots of positively tested peptides within the study cohort. Peptides defined as immunodominant (see Fig. 3a) are denoted by the first amino acid of the 15mer peptide used for single peptide testing. Amino acid positions of peptides within the protein sequence are indicated on the x-axis. Colored bars represent structural features of C and E as in Figure 3.
